# Metronidazole and Peripheral Neuropathy: A Report of Two Cases of (Unusual) Side Effects

**DOI:** 10.7759/cureus.30889

**Published:** 2022-10-31

**Authors:** Stefano Gussago, Cristiana Poroli Bastone, Diana Celio, Michele Arigoni, Massimo C Quarenghi

**Affiliations:** 1 General Surgery, Ospedale Regionale di Locarno, Ente Ospedaliero Cantonale, Locarno, CHE; 2 General and Visceral Surgery, Hôpital de Sion, Centre Hospitalier du Valais Romande, Sion, CHE; 3 Internal Medicine, Ospedale Regionale di Bellinzona e Valli, Ente Ospedaliero Cantonale, Bellinzona, CHE; 4 General Surgery, Regionalspital Uster, Uster, CHE; 5 Internal Medicine, Section of Clinical Nutrition and Dietetics, Ospedale Regionale di Bellinzona e Valli, Ente Ospedaliero Cantonale, Bellinzona, CHE

**Keywords:** side‐effect, alcohol intolerance, antibiotic, peripheral neuropathy, metronidazole

## Abstract

Metronidazole is an antibiotic commonly prescribed for anaerobic and protozoan infections. Despite its good safety profile, this drug frequently causes a series of well-known side effects (nausea and intestinal transit disorders, dysgeusia, headaches, and alcohol intolerance). However, there are few data in the literature, mainly case reports and case series, about the onset of peripheral neuropathy with a generally self-limiting course after drug withdrawal. Thus, we herein describe two cases of peripheral neuropathy due to treatment with metronidazole. A 69-year-old woman treated with a total of 55 g of metronidazole for diverticular disease and a 52-year-old male patient on a long course of antibiotic therapy for hepatic abscesses (a cumulative dose of 168 g) developed peripheral neuropathy. The suspicion of metronidazole side effects was raised after the exclusion of other causes. After the suspension of the drug, different degrees of improvement were observed. Metronidazole is an effective antibiotic for treating infections caused by anaerobic or protozoan pathogens, and it has a good pharmacological and economic safety profile. However, in the existing literature, prolonged therapy regimens (>4 weeks of treatment and/or 42 g cumulative dose) may increase the risk of developing neurological complications, in particular peripheral polyneuropathy.

## Introduction

In modern medicine, "side effect" is generically defined as any unintended or unwanted (not necessarily harmful) effect linked to the pharmacological action of a therapeutic substance [1]. These events are usually classified based on frequency according to the criteria of the Council for International Organizations of Medical Sciences: very common, common, uncommon, rare, and very rare [2].

In this regard, the most frequent extra neurologic side effects of metronidazole (1-(β-hydroxy-ethyl)-2-methyl- 5-nitroimidazole), an antibiotic commonly used for anaerobic and protozoan infections, are well known, including nausea and intestinal transit disorders, dysgeusia, headache, and alcohol intolerance (Antabuse effect) [3]. However, the occurrence of neurologic symptoms, particularly peripheral neuropathy that is prevalent in the lower limbs, during prolonged therapy with metronidazole is rarely reported [4,5].

Thus, we present herein two recently identified cases of peripheral neuropathy due to metronidazole treatment. Moreover, we also performed a brief review of the relevant existing literature.

## Case presentation

Clinical case 1

A 69-year-old woman with a generally good condition, who was on chronic steroid therapy for polymyalgia rheumatica and levothyroxine after subtotal thyroidectomy for Graves Basedow disease, presented to our institution due to a recurrence of acute diverticulitis of the sigma complicated by a covered perforation. A computed tomography scan shows a small abscess adjacent to the intermediate sigma (modified Hinchey Ib) [6] that is not appropriate for percutaneous drainage; hence, prolonged antibiotic therapy and subsequent surgical treatment by laparoscopic resection of the sigma were planned. Preoperatively, an antibiotic regime of ciprofloxacin (500 mg 2×/day) and metronidazole (500 mg 3×/day) was administered.

After the laparoscopic resection of the rectum sigma, antibiotic therapy is suspended. The immediate post-operative surgical course takes place without complications, with the rapid resumption of feeding and gastrointestinal transit. However, on day 5 after the surgery, the patient reports the appearance of allodynia and paresthesia in the lower limbs. A more in-depth anamnesis revealed that this symptomatology was already present, although of lesser severity, a few days prior to admission, and the progression of the disorder has greatly bothered the patient.

On neurological examination, the patient had dysesthesia with a bilaterally moderate pallesthesia, without significant trophic and stenic alterations, and with normoelicitable osteo-tendon reflexes. In particular, the patient has an unstable tightrope walk. No sphincter or urinary disorders were revealed on examination. Electroneurography does not show axonal or myelin abnormalities, consistent with polyneuropathy involving small fibers (Table 1).

**Table 1:** Electroneurography of the tibial and right peroneal nerves. msec: milliseconds; NCV: nerve conduction velocity.

Nerve	Neurography	Stimolus	Registration	Dist. (cm)	Latency (msec)	Answer	NCV (m/s)
Tibial right	Motoric	Internal malleolus	Abd. hallucis	12	5.0	12	
		Popliteal fossa	Abd. hallucis	39	13.0	12	48
		F wave			46.4		
Peroneal right	Motoric	Ankle	Ext. Dig. Brevis	6.5	4.5	8	
		Peroneal neck	Ext. Dig. Brevis	21	8.9	8	49
		Popliteal fossa	Ext. Dig. Brevis	10	10.9	8	49
H Reflex					29.4		
Sural right	Sensitive orthodromic			14	2.8	4 uV	50

A preoperative contrast-enhanced abdominal CT scan performed in the context of diverticular disease did not show any abnormalities of the lumbar spine. After ruling out diabetes, use of alcohol, and vitamin deficiency (in particular: cobalamin and folate), we attribute the origin to possible drug toxicity due to metronidazole administration. Reviewing the patient’s medical record, it is clear that during the numerous episodes of diverticulitis and recent conservative treatment, the patient has received a total of 55 g of metronidazole in the last eight weeks.

After metronidazole treatment was discontinued, it was possible in the following months to find a partial remission of the neurological problem with, however, a moderate residual lameness due to persistent hypoesthesia of the lower limbs.

Clinical case 2

A 52-year-old patient who, following a type A aortic dissection complicated by renal, hepatic, and intestinal ischemia, developed a type III short bowel syndrome [7] with partial dependence on parenteral nutrition, partial liver insufficiency, renal insufficiency, and chronic cholestasis due to a stenosis of the extra- and intrahepatic biliary tracts because of scarring.

The patient developed multiple polymicrobial hepatic abscesses (caused by *Enterococcus faecium* and *Escherichia coli*) due to recurrent cholangitis, which required multiple percutaneous drainages and retrograde endoscopic cholangio-pancreatography with biliary stent placement. The abscesses persisted despite repeated treatments; thus, a long-term antibiotic tri-therapy (amoxicillin 750 mg 3 ×/day, metronidazole 500 mg 3 ×/day, and ciprofloxacin 750 mg 2 ×/day) was initiated. After approximately four months of treatment, the patient develops a predominantly sensitive disorder, i.e., paresthesia and allodynia in the distal lower limb (especially the plantar region) and the left forearm with the sensation of pins and painful cramps.

During the consequent hospitalization, a slight motor impairment with disequilibrium and dysarthria was also observed; thus, the patient underwent magnetic resonance imaging (MRI) of the brain and epi-aortic vessels as well as a carotid echo-color-Doppler examination, which both showed negative findings. The most probable etiological hypothesis of the neurological symptoms seems to be related to a toxico-metabolic genesis; thus, further investigations for possible vitamin deficiencies (in particular: cobalamin, thiamine pyrophosphate, and pyridoxal-5-phosphate) or metal poisoning (e.g., copper, lead, and mercury) were performed, but no abnormalities were found (Table 2).

**Table 2:** Main biochemical investigations. IF, immunofluorescence; HIV, human immunodeficiency virus; HCV, hepatitis C virus; IgG, immunoglobulin G; IgM, immunoglobulin M; Ig, immunoglobulin.

Test	Value	Reference	Unit of measure
Copper	14.6	11.0–22.0	µmol/L
Zync	7.4	12.0–26.0	µmol/L
Selenium	0.76	0.64–1.52	µmol/L
Aluminum	0.30	<0.37	µmol/L
Lead	6	<10	nmol/L
Thiamine pyrophosphate	94	67–200	nmol/L
Pyridoxal-5-phosphate	103	35–110	nmol/L
Cobalamin	439	145–569	ρmol/L
Folic acid	34.8	8.8–60.8	nmol/L
Antinuclear antibodies IF	1/80	<1/80	-
Anti-neutrophil cytoplasmic antibodies	<1/80	<1/80	-
Anti-extractable nuclear antigen screen	Negative	Negative	-
Anti-neutrophil cytoplasmic antibodies IF	<1/10	<1/10	-
Rheumatoid factor	11.3	<15.0	U/mL
Anti-citrulline antibodies	0.8	<7–10	U/mL
Anti-neuronal antibodies (Hu, Ri)	<1/80	<1/80	-
Anti-Purkinje antibodies (Yo)	<1/80	<1/80	-
HIV antigen P-24	Negative	Negative	-
HIV IgG	Negative	Negative	-
HCV antibodies	Negative	Negative	-
Borrelia Burgdorferi IgG	0.14	<0.20	-
Borrelia Burgdorferi IgM	0.0	<0.2–0.32	-
Treponema Pallidum Ig	Negative	Negative	-

The diagnostic hypothesis, therefore, focuses on the potential toxic action of metronidazole; thus, after consulting with an infectious disease specialist, the metronidazole was suspended given the regression of liver abscesses on control MRI. Electromyoneurography revealed a moderate axonal sensorimotor-type polyneuropathy, consistent with a prolonged latency and smaller nerve conduction velocity, prevalent in the lower limbs (Tables 3-5).

**Table 3:** Electroneuromyography revealed a moderate axonal sensorimotor-type polyneuropathy, prevalent in the lower limbs. msec, milliseconds; NCV, nerve conduction velocity.

Nerve	Neurography	Stimolus	Registration	Distance (cm)	Latency (msec)	Potential (mV)	Duration (msec)	NCV (m/s)
Tibial right	Motoric	Abd. hallucis	Ankle	10.5	6.9	2.2	5.9	
		Ankle	Popliteal fossa	44.0	19.2	1.8	9.7	36
Peroneal right	Motoric	Ext. Dig. Brevis	Ankle	7.5	6.7	3.0	5.9	
		Ankle	Peroneal (head)	31.0	15.1	3.1	6.4	37
		Peroneal head	Popliteal fossa	8.0	18.0	2.8	6.4	28
Tibial left	Motoric	Abd. hallucis	Ankle	20.5	6.5	2.1	5.9	
		Ankle	Popliteal fossa	46.0	19.7	2.1	6.4	34
Peroneal left	Motoric	Ext. Dig. Brevis	Ankle	7.5	5.5	2.6	7.2	
		Ankle	Peroneal (head)	35.0	15.9	1.9	7.7	34
		Peroneal head	Popliteal fossa	10.0	17.9	2.1	7.8	50
Ulnar right	Motoric	Abductor digiti minimi	Wrist	7.5	3.2	7.3	7.0	
		Wrist	Distal solcus	26.0	8.3	7.2	6.9	51
		Distal solcus	Proximal solcus	20.0	10.5	6.6	7.7	45
Sural right	Sensitive					Absent		
Sural left	Sensitive					Absent		
Ulnar right	Sensitive					Absent		

**Table 4:** Needle electromyography.

Measurement site	Insertion	Spontaneous activity			Voluntary activity			Maximum effort	
	Duration	Positive waves	Fibrillation	Fasciculation	Amplitude	Duration	Polyphasic	Trace	Effort
Gastrocnemius medial right	-	-	-	-	Increased	Normal	Normal	Decreased	

**Table 5:** Motor unit analysis at gastrocnemius medial right. MUP, motor unit potential.

Motor Unit potential records	Duration	Amplitude	Phases	Duration spikes
Type of MPU	Number of MUP: 1		Polyphasic MUP: 0%	
Medium value of all MUP	10.9 msec	3245 µV	3.0	8.5 msec
Medium value of not polyphasic MUP	10.9 msec	3245 µV	3.0	8.5 msec

To determine the etiology, we also performed an investigation on auto-antibodies [antinuclear antibodies, extractable nuclear antigen, anti-neutrophil cytoplasmic antibodies, anti-citrulline, rheumatoid factor, anti-neuronal antibodies (Hu, Ri), and anti-Purkinje (YO)] and antibodies for HIV, hepatitis C virus, Borrelia burgdorferi, and Treponema pallidum, which showed no abnormal results (Table 2).

The patient had rapid improvement of dysarthria (one week) after discontinuation of antibiotic therapy with metronidazole. Moreover, progressive improvement of the peripheral neuropathy was observed, despite the presence of residual bilateral stocking hypoesthesia of the lower limbs and the distal phalanges of both hands, at <4 months after the suspension of therapy.

## Discussion

In the existing literature [4,5,8], the appearance of peripheral neuropathy is described as among the potential side effects of metronidazole, but its exact prevalence and cumulative doses are not well established. Neither a clear pathophysiological mechanism of peripheral neuropathy related to metronidazole is established, although some role seems to be played by the toxicity of its metabolites [5].

We report two cases of peripheral neuropathy that, after extensive etiological investigation, were attributed to a side effect of metronidazole occurring in two patients with very different manifestations: a woman without a marked medical history and a man with hepato-renal insufficiency. Peripheral neuropathy, as shown by the two electroneuromyography studies (Tables 1-3), can be attributed to different mechanisms. In case 1, the patient presents with an isolated peripheral neuropathy resulting probably from damage involving the small fibers, such as the case has been described in previous case reports [4], whereas case 2 presents a mixed disorder with both central (considering the dysarthria) and peripheral symptoms. In this latter, the patient had a symmetric moderate axonal sensorimotor-type polyneuropathy documented at the electromyoneurography. These findings are consistent with previous literature, where metronidazole-induced paresthesia is linked to predominant small fiber involvement, although, to a lesser extent, large fibers may also be involved [9]. Interestingly, despite the involvement of the central nervous system, the second patient had normal MRI findings with no evidence of cerebellar abnormalities, as described in some case reports [10,11].

In 2013, in a randomized trial conducted by Carroll et al. to evaluate the efficacy of oral metronidazole therapy (500 mg 3 ×/day) for tuberculosis pneumonia caused by multi-resistant germs, the therapy was suspended in advance owing to a 4.3-fold higher risk of developing peripheral neuropathies after six months of treatment in the treatment group (almost 50% of patients) than in the placebo arm [12].

In 2010, the Society for Healthcare Epidemiology of America and the Infectious Diseases Society of America, which provided the guidelines for treating Clostridium difficile infections (grade IIB), recommended that metronidazole should not be used to treat recurrences or as a long-term treatment owing to the risk of developing cumulative neurotoxicity [13].

The recent review by Goolsby et al. in 2018 [5], which included 13 clinical studies and >36 case reports, described that the actual incidence of this side effect in patients receiving treatment with metronidazole is still controversial, with the studies showing extremely wide variations (0-50%). However, this complication appears to be associated with prolonged therapy, i.e., generally >4 weeks and with a cumulative metronidazole dose >42 g, with the risk of developing peripheral neuropathy during metronidazole treatment rising from 1.7% to 17.9% in the latter subgroup [5]. The cumulative dose of case 1 was 55 g within eight weeks of treatment and that of case 2 was approximately 168 g within 16 weeks of treatment before the first symptoms of neuropathy appeared. On the other hand, Daneman et al. found no correlation between the cumulative dose and the occurrence of neuropathy [14].

It, therefore, remains imperative to suspend treatment with metronidazole in the event of suspected peripheral neuropathy. Moreover, evidence from the literature indicates that a restitutio ad integrum is often obtained, even if the timing for complete resolution, if reached, is very variable (from 2 weeks to 1.5 years) [5]. In case 1, improvement of the neuropathy symptoms improved two months after treatment suspension, whereas in case 2, partial regression of symptoms was observed at four months after treatment suspension, although residual acral hypoesthesia in the lower limbs still persisted.

Although the abovementioned evidence may discourage the use of metronidazole, we must remember that, generally, this drug is well tolerated; in monotherapy and in regimens of poly antibiotic therapy, the cumulative dose is lower than that considered risky in the literature. A classic therapy of 7-10 days as indicated, for example, in the case of uncomplicated diverticulitis [15], results in a cumulative dose of 14-20 g, which is well below the dose reported by Goolsby et al. in their review [5].

Furthermore, the spectrum of anti-anaerobic antimicrobial activity makes metronidazole a good drug for controlling diseases such as intra-abdominal infections [3], for which the therapeutic alternatives are generally more expensive, and whose abuse could lead to an increase in bacteriological resistance.

## Conclusions

Metronidazole is an effective antibiotic for treating infections caused by anaerobic or protozoan pathogens, and it has a good pharmacological and economic safety profile. However, in the existing literature, prolonged therapy regimens (>4 weeks of treatment and 42 g cumulative dose) may increase the risk of developing neurological complications, in particular peripheral polyneuropathy. Thus, it is advisable that, if a prolonged therapeutic regimen is prescribed, the appearance of peripheral neurological symptoms should be monitored; if such cases occur, it is mandatory to suspend the therapy. In patients with peripheral neurological symptoms, the prognosis is generally favorable with complete remission of the symptomatology in the majority of cases after treatment suspension, although, in the literature, the timing for a restitutio ad integrum results extremely varies.
